# Clinical Indicator Implementation in Japanese Public Hospitals: A Comparative Study of the 2016 and 2024 Trends After the COVID-19 Pandemic

**DOI:** 10.7759/cureus.88676

**Published:** 2025-07-24

**Authors:** Yasutoshi Moteki

**Affiliations:** 1 Health Policy, Faculty of Policy Studies, Nanzan University, Nagoya, JPN

**Keywords:** clinical indicators, clinical pathway, hospital indicators, japan, medical quality

## Abstract

Aim

This study investigates the adoption and implementation of clinical indicators in Japanese public hospitals, focusing on changes since the coronavirus disease 2019 (COVID-19) pandemic and utilizing Donabedian’s triadic model (structure, process, outcome).

Methods

A nationwide postal survey was conducted targeting 848 municipal hospitals with ≥20 beds in Japan. The survey explored clinical indicator usage, categorized by Donabedian’s framework. Hospitals were classified based on size to evaluate disparities in indicator adoption. Data were analyzed to compare findings from the 2024 survey with a similar 2016 survey. Questionnaires were mailed at the end of September 2024. Survey participants were provided with an extended deadline of December 16, 2024, which was one month after the original deadline.

Results

As of December 17, 2024, the response rate was 15.7% (133 hospitals), with 130 valid responses. Clinical indicator adoption increased from 42.4% (97) (2016) to 58.5% (76) (2024). Large hospitals were more likely to implement clinical indicators (59 [88.1%]) than small and medium hospitals (17 [27.0%]). Frequently used indicators included average hospitalization duration, bed utilization rates, and clinical pathway coverage. Each structural indicator remained underutilized, with <15% of hospitals reporting their use.

Conclusion

The findings highlight progress in clinical indicator adoption, particularly in large hospitals. However, significant disparities persist between hospital sizes. As the pandemic subsides, it remains essential to continue improving hospital management practices, including clinical indicator use, in preparation for future infectious disease outbreaks. The three-way model classification for medical evaluation is renowned, but the percentage use of structural indicators has not exceeded 15%, and the implementation status is not as advanced as that of process and outcome indicators. Our results are significant as they verify the explanatory power of the three-way model for medical care based on Japanese data.

## Introduction

The spread of coronavirus disease 2019 (COVID-19) in Japan prompted the central government to declare a state of emergency in specific regions four times, from April 2020 to September 2021, based on the Act on Special Measures for Pandemic Influenza and New Infectious Diseases Preparedness and Response (Act No. 31 of 2012) [1]. Public hospitals operated by local governments were central to the hospital-level response to the outbreak as a designated medical institution for infectious disease [2]. Specifically, these hospitals played a pivotal role in outpatient responses and inpatient treatment for COVID-19. This role stems from the historical function of public hospitals in Japan, which includes infectious disease control, remote medical care, and care for individuals with disabilities; these services are often unprofitable for private hospitals [3]. Local governments have maintained these essential medical functions by closely managing public hospital operations. In a broad context, the term “public hospitals” encompasses state-run hospitals and those affiliated with national universities. However, in the present study, this term is used exclusively to refer to municipal public hospitals. Under this definition, the operational characteristics of Japanese public hospitals include their significant role in addressing unprofitable areas of public interest that are not financially viable for private hospitals to manage. These areas include treatment of infectious diseases, medical care for individuals with disabilities, healthcare in remote regions, and emergency medical services. In the event of an operational deficit, funds may be allocated from the general account budget of the operating municipality provided that certain conditions are met.

Japan's public health insurance system, which was introduced based on the Health Insurance Law of 1922 and was established under the National Health Insurance Law of 1958, has been instrumental in delivering healthcare services and achieving remarkable health outcomes. Despite facing financial difficulties during the Great Depression at the end of the 1920s, the system stabilized with economic growth [4]. Universal healthcare coverage through public health insurance with fee-for-service payment has ensured equitable distribution of health services and reduced the burden on families providing care for older adults [4]. While public hospitals have been critical to Japan's healthcare system, private sector hospitals dominate, particularly in mental healthcare. Over 80% of psychiatric beds are privately owned, and private hospitals serve all citizens covered by the universal public health insurance system [5]. This contrasts with many Western countries and reflects a unique aspect of Japan's healthcare system. Combining public insurance with a mix of public and private providers, Japan's healthcare system has evolved into one of the world's most effective [6]. Nonetheless, challenges persist, particularly in mental healthcare, which remains inpatient-focused despite efforts to shift toward community-based care [5]. The government primarily acts as a regulator, while service provision is largely managed by the private sector [7], creating a unique balance between public and private involvement in healthcare delivery.

During the COVID-19 pandemic, Japanese public hospitals implemented various strategies to manage the crisis and maintain healthcare access. Research highlights that public hospitals played a central role in responding to COVID-19 infections, which disrupted the medical functions they typically performed during non-pandemic periods. The first wave of the pandemic in Japan caused a nationwide decrease in outpatient visits and hospitalizations, particularly for long-term care beds [8]. This underscored the need for long-term health monitoring of vulnerable populations and urgent support for public healthcare facilities to prepare for prolonged pandemics. Ishii et al. studied the pandemic’s impact on a specific medical field [9]. Despite challenges, most Japanese institutions continued primary percutaneous coronary interventions (PCIs) for ST elevation myocardial infarction (STEMI) patients during the second and third waves. Over time, hospitals reported increased availability of personal protective equipment and more frequent COVID-19 screenings for patients.

As of May 8, 2023, new coronavirus infection was classified as a “Class 5 Infectious Disease” in Japan, with special measures largely discontinued at medical institutions. Japan's healthcare system demonstrated resilience during the pandemic through strategies, such as stratified patient care, dedicated COVID-19 hospitals and emergency centers, and respiratory care split hospitals, to mitigate community transmission and prevent nosocomial infections. Honda et al. conducted a detailed study on the coordination of COVID-19 responses and routine care at two public hospitals [10].

Even before the COVID-19 outbreak, Japanese public hospitals were responsible for medical care classified as a public good - a field that was difficult for private hospitals to handle. Despite the subsiding of the pandemic, the financial situation of Japanese public hospitals remains challenging. Until FY2022, when public hospitals were addressing coronavirus infections, their financial performance had been improving owing to government subsidies for coronavirus countermeasures. However, in FY2023, as these coronavirus countermeasures were scaled back and government subsidies were reduced, the total deficit for the national public hospital sector amounted to 2055 billion yen [11]. In FY2022, there was a surplus of ¥199.6 billion, according to this newspaper article. Once again, the management situation of Japanese public hospitals became more challenging. In an effort to improve operational efficiency, Japanese public hospitals implemented various initiatives. For example, they promoted group purchasing of pharmaceuticals to capitalize on economies of scale and enhance bargaining power [12]. However, research shows that hospital scale does not significantly improve bargaining power in drug purchasing. Instead, assigning management responsibility for economic efficiency to public hospitals has proven to be a more effective strategy [12]. In Japan, efforts to improve hospital management from a management engineering perspective have long focused on the “quality of medical care,” including public hospitals. Statistical quality control (QC) and other management engineering initiatives were promoted through collaboration between business and academia during Japan’s post-World War II economic recovery. QC, developed by Nikkagiren - a foundation uniting Japanese industry, academia, and government - aimed to enhance and optimize production management in private enterprises using scientific approaches such as statistics for industrial advancement. QC emphasizes workplace discussions and small-group suggestions for production improvements, known as QC circles. These circles gained international attention during Japan’s economic boom, with some corporations adopting the practice. QC refers to both quality control and quality circles. Toyota Motor Corporation's QC practice, Kaizen, has achieved global recognition for its application in corporate management and research. This method is often called total quality control but has since become part of a UK-based academic journal (The TQM Journal) and is now globally referred to as total quality management (TQM). In Japan, Nikkagiren began using the term TQM in 1996 [13]. Although QC activities originated in the factory sector, similar management engineering initiatives have expanded to hospitals, schools, and other service industries.

This focus on “quality of care” is closely linked to the management challenges faced by public hospitals and has driven initiatives, such as the adoption of clinical indicators and other private-sector management techniques. Palma et al. examined the quality of the oncology health service in a public hospital in Italy using structural equation models by referring to the SERVQUAL scale that began to be used in the private sector management field after the 1980s [14]. Moteki examined the status of clinical indicator implementation in public hospitals as of 2016 through a questionnaire survey [15]. This paper reported on the use of clinical indicators in 2024, eight years later, following the completion of the COVID-19 response described above, and compared the findings with the previous study using the same survey tool.

However, in the global context, Donabedian's triadic theory is highly recognized for evaluating medical care [16]. Donabedian’s framework, encompassing structure, process, and outcome, has been widely applied in healthcare quality assessments across various settings. Research has explored clinical indicators in multiple medical specialties. For instance, Haller et al. conducted a systematic review to assess quality and safety indicators in anesthesia [17], while Hamilton-Davies et al. compared clinical indicators for hypovolemia [18]. Donabedian’s model offers a systematic method to evaluate and improve healthcare quality [19,20]. It has been applied to diverse areas, including systemic lupus erythematosus care, emergency medical services, mental healthcare for veterans, and hospice programs [21-23]. Some researchers have proposed extensions to Donabedian’s model. For example, Begicheva proposed adding a fourth category, environmental quality, to address the unique needs of emergency medical services in large metropolitan areas [21]. Mitchell et al. presented a dynamic model that introduces reciprocal relationships between components, moving beyond Donabedian's linear framework [20]. Additionally, Hynes and Thomas suggested integrating Donabedian’s framework with the Andersen individual behavioral model to enhance understanding of care coordination in healthcare [24]. Donabedian's framework remains a vital tool for assessing and improving healthcare quality. Its applications extend beyond traditional healthcare settings to fields such as education [25] and modeling of healthcare systems using cases obtained from the Italian healthcare services [26]. However, as healthcare evolves, researchers continue to adapt and refine the framework to address emerging challenges and complexities in healthcare delivery and quality assessment [27,28]. These adaptations reflect the enduring relevance of Donabedian’s framework while underscoring the need for ongoing updates to meet the dynamic demands of healthcare quality evaluation.

This study surveyed public hospitals across Japan to analyze issues and challenges related to clinical indicators. Questionnaires were sent to all 848 municipal hospitals with 20+ beds. The 2016 survey, conducted jointly with another researcher, included questions about privacy practices in public hospitals. Results from the privacy protection portion of that survey were reported by Hashimoto and Moteki [29], while the analysis of clinical indicators is detailed by Moteki [15]. This study compares changes in the utilization of clinical indicators post-pandemic. The present survey, conducted solely by the author, focused exclusively on the implementation of clinical indicators. It included questions about clinical indicators used as performance measures for hospital activities and open-ended questions about practical challenges and issues in managing personal information. The objectives of this study were to examine changes in the operational status of clinical indicators in public hospitals following the response to COVID-19 and to evaluate the current status of clinical indicators in Japan using Donabedian’s three-way model, which is an internationally recognized framework that incorporates structure, process, and outcome to evaluate healthcare. In other words, the main aim of this study was to grasp the actual operation of clinical indicators and their changes based on two surveys in the Japanese context and to theoretically test the validity of Donabedian's three-way model.

Related studies

Numerous studies on clinical indicators fall into the following three categories: (1) literature examining clinical indicators in hospitals to improve organizational management within the medical field, (2) literature focusing on clinical indicators in specific disease areas, and (3) studies on clinical indicators in Japan. Clinical indicators are essential tools for assessing and improving healthcare quality, offering a quantitative basis for evaluating structures, processes, and outcomes [30]. These indicators can be rate-based, mean-based, or sentinel and may be generic or disease-specific [30]. Their use in routine practice reflects clinical care processes and improvements in outcomes. For example, in inflammatory arthritis management, quality indicators span multiple domains, including structure, process, patient experience, outcome, access, and efficiency [31], allowing for a holistic assessment of care quality. While clinical indicators are crucial for quality improvement, their implementation poses challenges. In graduate medical education, defining and measuring clinical performance can be complex, as can using electronic health records and clinical registry data to capture such information [32]. Similarly, patient safety indicators in high-risk surgeries, such as cranial neurosurgery, show higher risk-adjusted rates than those in other surgeries, emphasizing the need for caution when applying these metrics to quality improvement or pay-for-performance systems [33]. Public hospitals use clinical indicators to evaluate quality, efficiency, and performance, focusing on areas such as patient safety, clinical outcomes, and service delivery. Examples include hospital infection rates, hospital accident prevalence, and hospital mortality rates [34]. However, the ease of measurement varies significantly across indicators, with only three out of 52 key performance indicators identified as "easily measurable" in one study [35].

Regarding the use of clinical indicators in specific disease areas, Ballmer et al. conducted a scoping review of quality indicators in occupational therapy [36]. Their findings classified indicators into process level, functional outcome, and individual goal attainment indicators (e.g., the Goal Attainment Scale), along with PRO-Ergo, a patient-reported experience measure. A similar review by Hadian et al. categorized hospital indicators into three main groups, 14 subcategories, 21 performance dimensions, and 110 primary indicators, further grouping them by input, process, output, outcome, and impact [37]. Haller et al. explored a set of standardized and valid clinical outcome indicators for use in perioperative clinical trials in the field of anesthesiology based on Donabedian’s theory [38]. Based on video data from 87 surgical cases, Kaoukabani et al. found a relationship between clinical performance indicators and surgical characteristics (surgical experience, case complexity, etc.) in robot-assisted cholecystectomy [39]. Kieft et al. explored nurse-sensitive indicators (NSIs) in German hospitals, focusing on areas such as pain, wound care, malnutrition, and delirium [40]. Their study revealed insufficient scientific evidence supporting these indicators, highlighting methodological challenges in NSIs for the nursing field. Similarly, Allen et al. surveyed 176 obstetrics and gynecology facilities in England, examining whether performance indicators developed by the Royal College of Obstetricians and Gynaecologists aligned with Care Quality Commission ratings [41]. They found no statistically significant relationships between inspection ratings and performance indicators in maternity services, raising questions about the validity and reliability of both metrics.

In Japan, limited studies address the use of clinical indicators in hospitals. Notable exceptions include a study by Furuhata et al. [42], who statistically confirmed the impact of introducing clinical pathways, such as shorter hospital stay, using data from 6,523 patients at Miyazaki University Hospital. Similarly, other literature explores clinical indicators in specific medical fields in Japan [43,44]. Miyazaki et al. empirically examined 980 subacute stroke patients to assess the utility of clinical indices [43]. Focusing on Japanese public hospitals, the author's study examines the introduction and changes in clinical indicators over time to improve management from a management engineering perspective. No other study has explored this specific field or analyzed the data longitudinally, underscoring the novelty and uniqueness of this research.

## Materials and methods

To investigate challenges associated with the use of clinical indicators in Japanese medical facilities operated by local governments, a postal survey was distributed to public hospitals nationwide. The focus on public hospitals stemmed from their significant contribution to public healthcare, particularly in responding to the new coronavirus infection, compared to private institutions. Questionnaires were sent to 848 hospitals with ≥20 beds, all of which were members of the Japan Municipal Hospital Association (JMHA). The survey targeted hospitals with ≥20 beds, identified from the JMHA member facility database as of April 1, 2024 [45]. Questionnaires were mailed at the end of September 2024. Shortly thereafter, it was confirmed that Higashi Matsudo Hospital had closed on March 31, 2024, as the questionnaire was returned undelivered with the label “Return Unknown.” Accordingly, the population of municipal hospitals surveyed was 847.

The questionnaire included the same questions as those included in the previous 2016 survey [29]. The previous survey included questions about the management of patients’ personal information; however, the 2024 survey only included questions about clinical indicators. The results of the clinical indicators portion of the 2016 survey are available elsewhere [15]. The 2024 survey comprised 11 questions. Question 1 (Q1) was a so-called fact sheet about the responding hospitals, and question 2 (Q2) through question 7 (Q7) were optional questions about the implementation of clinical indicators. Question 8 (Q8) through question 11 (Q11) allowed open-ended responses regarding the implementation of clinical indicators and this survey. This open-ended portion was not directly discussed in this work. Question 6 (Q6) and Q7 were new additions to the 2024 survey and asked about the individual implementation of 56 clinical indicators and 7 hospital indicators, respectively. All responses to Q6 are presented in the Appendix. Examples of clinical indicators discussed in Q6 and their respective structures, results, and process classifications were based on examples of clinical indicators implemented at Shizuoka Cancer Center [46].

The survey request was addressed to hospital departments responsible for medical QC, which were tasked with completing the questionnaires. Participants were assured that survey data would be aggregated and analyzed, with results published, but that individual hospital responses would not be disclosed in their original form. Contact information for the researchers was provided for inquiries. Follow-up reminders were sent to non-responding hospitals after the initial submission deadline. The reminder set an extended deadline of December 16, 2024, one month after the original deadline. As of December 17, 2024, the response rate was 15.7% (133 hospitals). Among these, three hospitals returned the questionnaire but did not answer individual questions. The final number of valid responses was, therefore, 130 hospitals. The previous response rate was 26.6%. This study offers valuable insights into Japanese public hospitals prior to the COVID-19 pandemic.

This article was previously posted to the Research Square preprint server on February 18, 2025.

## Results

Table 1 presents the bed capacity of responding municipal hospitals as of April 1, 2024. Valid responses were obtained from 130 municipal hospitals, ranging from large hospitals with >900 beds to small hospitals with <100 beds. The most frequent category comprised hospitals with <100 beds, followed by those with 100-199 beds. Municipal hospitals with ≥800 beds were rare. The number of full-time physicians was another measure of hospital size, with a median of 21.5, an average of 62.4, and a standard deviation of 90.5. For the subsequent analysis, hospitals were classified as "small and medium" if they had <200 beds and as "large hospitals" if they had ≥200 beds.

**Table 1:** Number of beds (Q1)

Number of beds	Count	Ratio
900–999	1	0.8%
800–899	1	0.8%
700–799	1	0.8%
600–699	11	8.5%
500–599	13	10.0%
400–499	12	9.2%
300–399	15	11.5%
200–299	13	10.0%
100–199	30	23.1%
<100	33	25.4%
Total	130	100.0%

Table 2 illustrates the correlation between hospital size and the measurement of clinical indicators. Among the responding hospitals, 58.5% (76) had adopted clinical indicator measurement, an increase of approximately 14 percentage points from 42.4% (97) in the 2016 survey. Over 80% of large hospitals utilized these metrics, compared to only 27.0% (17) of small and medium-sized facilities. Table 3 elaborates on this disparity, revealing that >70% of large hospitals published their clinical indicators online, compared to <22% of smaller institutions (The total of 86 in this table is the number of hospitals implementing the clinical indicator among the responses.). This discrepancy could stem from website-related limitations in smaller hospitals. Table 4 shows the relationship between clinical and hospital indicators. Of the 130 hospitals, 57 employed both clinical and hospital indicators, while 49 used neither. There is a strong association between the adoption of these two indicators. Hospital indicators, which pertain to hospital management, are uniformly measured using diagnosis procedure combination (DPC) data based on the DPC system, predominantly applied in larger hospitals. In this questionnaire survey, Q7 assessed the measurement status of seven DPC-based hospital indicators. Almost all hospitals covered by the DPC system circled all seven indicators.

**Table 2:** Use of clinical indicators according to hospital size

Hospital size	Implementing	Not implementing	Total
Small and medium	17 (27.0%)	46 (73.0%)	63 (100.0%)
Large	59 (88.1%)	8 (11.9%)	67 (100.0%)
Total	76	54	130

**Table 3:** Hospitals that published data on clinical indicators online versus those that did not Note: The total is 80 and exceeds 76 because there are several cases where Q3 is 2 (not introduced), but the respondent answered 2 (not publicly disclosed) in sub-question 1.

Hospital size	Publishing on the hospital website	Not publishing on the hospital website	Total
Small and medium	6 (31.6%)	13 (68.4%)	19 (100.0%)
Large	48 (78.7%)	13 (21.3%)	61 (100.0%)
Total	54	26	80

**Table 4:** Comparison of hospital indicator use

	Implementing clinical indicators	Not implementing clinical indicators	Total
Implementing hospital indicators	57 (91.9%)	5 (8.1%)	62 (100.0%)
Not implementing hospital indicators	19 (27.9%)	49 (72.1%)	68 (100.0%)
Total	76	54	130

Table 5 outlines the prevalence of specific clinical indicators among hospitals, comparing the results of 2024 with those of 2016. The most commonly tracked metrics were average hospitalization duration and bed utilization rate, measured by 68.4% of hospitals. In the 2016 survey, this figure was 80.4%, reflecting a 12-percentage-point decrease. Clinical path coverage rates and ambulance reception/refusal rates were tracked by >50% of public hospitals using clinical indicators, a nearly 10-percentage-point increase compared to 2016.

**Table 5:** Measured clinical indicators Note: The initial numbering at the beginning of the indicator name corresponds to the Appendix’s indicator reference number.

Clinical indicators	2024 survey		2016 survey	
	Measuring	% of total	Measuring	% of total
A-1, Average length of hospitalization and rate of hospital bed utilization	52	68.4%	78	80.4%
A-2, Completion rate of intake summary	11	14.5%	34	35.1%
A-7, Coverage rate of clinical pathway	40	52.6%	42	43.3%
A-8, Average length of hospital stay for patients with cerebral infarction	11	14.5%	12	12.4%
A-9, Average length of hospital stay for patients with acute myocardial infarction	3	3.9%	8	8.2%
A-10, Diabetes: HbA1c improvement rate and the number of referrals and reverse referrals of patients with diabetes	1	1.3%	5	5.2%
A-11, Pneumonia: Average length of stay and success rate of initial treatment	1	1.3%	4	4.1%
D-1, Five-year survival rate after cancer surgery	4	5.3%	10	10.3%
D-2, Post-operative hospital stay for gastrointestinal cancer patients	1	1.3%	9	9.3%
D-3, Post-operative hospital stay for lung cancer patients	2	2.6%	8	8.2%
Proportion of breast-conserving surgeries in breast cancer patients	-	-	18	18.6%
A-12, Number of ambulances received and refused (rate)	31	40.8%	39	40.2%
A-13, Transport of pregnant women: number of admissions and refusals (rate)	5	6.6%	4	4.1%
A-14, Rehabilitation: early recommendation rate for rehabilitation within 2 days after hospitalization	5	6.6%	8	8.2%
I-6, Medical social workers: rates of medical social worker intervention for patients transferred to other hospitals and institutions	4	5.3%	9	9.3%
L-4, Number of supervisors per resident doctor	6	7.9%	8	8.2%
J-1, Percentage of respondents who would recommend the hospital to their friends in a patient survey	17	22.4%	13	13.4%
J-2, Percentage of ambulant patients waiting for at least 1 hour before seeing a doctor	8	10.5%	11	11.3%
Other	-	-	29	29.9%

The Appendix presents the results of clinical indicator implementation across the areas addressed in Q6 of the questionnaire. Alongside the number and percentage of hospitals measuring each indicator, the table includes its classification within the Donabedian three-way model and whether it qualifies as a clinical indicator as defined by the JMHA. The classification under the Donabedian ternary model follows the framework used by the Shizuoka Cancer Center, which served as a reference during the questionnaire's development. Examining the implementation status of clinical indicators based on their position in Donabedian’s three-way model reveals that most indicators with high implementation rates are categorized under outcome and process. However, the number of indicators classified under structure is small, with only three items included in the current questionnaire. Moreover, the percentage of each indicator remains <15%. The survey results highlight that the percentage of indicators classified under structure is significantly less advanced than those under outcome and process.

## Discussion

The Donabedian model is renowned for the assessment of healthcare quality, including clinical indicators [16]. The Donabedian model proposed three aspects for evaluating healthcare quality: structure, process, and outcomes. Donabedian’s three-way model is frequently referenced and analyzed in the evaluation of Japan’s healthcare sector. However, in certain areas, Japan’s healthcare evaluation has evolved independently, focusing on “quality of medical care” from a management engineering perspective, incorporating methods such as TQM and clinical pathways, and emphasizing medical safety. The findings of this study revealed that indicators categorized under structure have an adoption rate of ≤15%, with implementation less advanced compared to outcome and process indicators. Since this study focuses exclusively on Japanese hospitals, it remains unclear whether the limited progress in adopting structure indicators is unique to Japan or represents a global trend. Therefore, one study that reported statistically significant correlations between structure and process indicators (r=0.33) and between process and outcome indicators (readmission r=−0.33; length of stay r=−0.27) of an integrated trauma care system has gained attention [47]. As Donabedian argued, if the appropriate structure is not ensured, then the subsequent processes and outcomes related to quality of care are not ensured. Further development of international comparative studies examining the relevance of the three-way model classification, in conjunction with this study, could enhance the theoretical frameworks of medical evaluation.

This study compared the results of a 2024 survey on clinical indicator assessment in Japanese public hospitals with the findings of the 2016 survey questionnaire by Moteki [15]. The current results revealed significant differences between large and small/medium hospitals in their use of clinical indicators (Table 2). Nearly 30% of small and medium hospitals adopted these metrics, highlighting notable variation based on hospital size. The survey also analyzed secular changes in the adoption of specific clinical indicators (Table 5). Average hospitalization duration and bed utilization rate were the most frequently measured indicators (68.4%). “Clinical path coverage rate” and "number of ambulances received and refused (rate)" exceeded 40% usage, while other indicators were reported by fewer than 40% of respondents. Notably, the post-pandemic survey revealed a 10-point decline in the use of average hospitalization duration and bed utilization rate as indicators. Conversely, the adoption of clinical pathway coverage increased by approximately 10 percentage points. Regarding the former, the response to the novel coronavirus infection, which significantly impacted Japan in 2020-2021, may have influenced changes in hospital bed utilization management. In other words, traditionally, a high bed utilization ratio has been considered fundamentally desirable for maximizing hospital facility efficiency. However, during the pandemic, hospitals had to manage both routine care and admissions of patients with infectious diseases. As a result, maintaining a margin of bed utilization became essential for effective hospital operational management. It is assumed that this change in the meaning of bed utilization rate has been partly responsible for the decline in the percentage of bed utilization used as a clinical indicator in Japanese public hospitals.

Limitations and future research agendas

The primary limitation of the findings in this paper is the decline in response rate since the 2016 survey. Alongside the deteriorating financial conditions of Japan's local governments, the management of public hospitals run by these entities is also worsening. Due to privatization and hospital mergers, the survey population decreased from 887 to 848. Additionally, telephone calls and returned survey forms indicated a lack of surplus capacity to respond to the questionnaire due to administrative burdens. The worsening operational severity of public hospitals likely contributed to lower response rates.

Notably, the survey results captured changes over time, as shown in Table 5 and elsewhere, compared to the 2016 survey. We plan to conduct another questionnaire survey on clinical indicators in Japanese public hospitals in 8-10 years, as done in this study. To improve the collection rate, we aim to focus on the most frequently used indicators and reduce the burden on respondents completing the questionnaire. By implementing these changes, we hope to conduct another similar survey to identify changes and trends in the use of clinical indicators in Japanese public hospitals over time, ultimately contributing to international research on healthcare management. Canning et al. have conducted a study defining quality indicators for healthcare by applying a four-round modified-Delphi approach from an inductive standpoint, unlike Donabedian’s deductive three-way model [48]. As was attempted in this study, in the future, it will be necessary to empirically explore a set of clinical indicators that are appropriate for each medical field and setting without assuming the three-way model of Donabedian’s evaluation of medical care, which is our future research agenda.

## Conclusions

International research on clinical indicator implementation in public hospitals is advancing, with studies reporting average values for specific indicators based on surveys. This research highlights the status of clinical indicator implementation in Japanese public hospitals and the adoption rate of each indicator, aspects not previously examined in English language literature. Municipal hospitals vary in their departments, locations, and establishment purposes. The 2016 survey data predate the COVID-19 pandemic in Japan. The findings highlight progress in the adoption of clinical indicators, particularly in large hospitals. However, significant disparities among hospital sizes persist. As the pandemic subsides, continuous improvement of hospital management practices, including clinical indicator use, remains essential in preparation for future infectious disease outbreaks. The three-way model classification for medical evaluation is renowned, but the percentage use of structural indicators has not exceeded 15%, and the implementation status is not as advanced as that of process and outcome indicators. Our results are important because they verify the explanatory power of the three-way model for medical care based on Japanese data.

## Appendices

**Table 6:** Appendix classification and adoption rate of each clinical indicator Note: X means applicable, i.e., consistent with the indicators set by the Japan Municipal Hospital Association. MSW, medical social worker

Indicator Reference Number	Name of Clinical Indicator	Structures, Outcomes, and Processes	Indicators Proposed by the Japan Municipal Hospital Association in Their Program	Number of Hospitals Measuring Each Indicator, N (%)
A: Hospital-wide initiatives and individual disease-related initiatives				
A-1	Average length of hospitalization and rate of hospital bed utilization	-	-	52 (68.4%)
A-2	Completion rate of intake summary	-	-	11 (14.5%)
A-3	Rate of unscheduled and emergency readmissions within 7 days of discharge	Outcome	-	14 (18.4%)
A-4	Unscheduled readmission rate within 30 days of discharge	Outcome	-	18 (23.7%)
A-5	Critical illness, medical, and nursing care needs in general wards	Process	-	18 (23.7%)
A-6	Discharge summary completion rate within 14 days of discharge	Process	-	23 (30.3%)
A-7	Rate of patients on clinical (critical) paths among inpatients	-	X	40 (52.6%)
A-8	Average length of hospital stay for cerebral infarction patients	-	-	11 (14.5%)
A-9	Average length of hospital stay for patients with acute myocardial infarction	-	-	3 (3.9%)
A-10	Diabetes: HbA1c improvement rate and the number of referrals and reverse referrals of patients with diabetes	-	-	1 (1.3%)
A-11	Pneumonia: Average length of stay and success rate of initial treatment	-	-	1 (1.3%)
A-12	Number of ambulances received and refused (rate)	-	-	31 (40.8%)
A-13	Transport of pregnant females: Number of admissions and refusals (rate)	-	-	5 (6.6%)
A-14	Rehabilitation: early recommendation rate for rehabilitation within 2 days after hospitalization	-	-	5 (6.6%)
B: Medical safety				
B-1	Falls and falls incidence in hospitalized patients	Outcome	X	63 (82.9%)
B-2	Falls and falls incidence in inpatients aged ≥65 years	Outcome	X	24 (31.6%)
B-3	Number of incident and accident reports	Outcome	-	45 (59.2%)
B-4	Percentage of all reports made by physicians	Outcome	-	29 (38.2%)
B-5	Rate of drug administration guidance (inpatients)	Process	X	28 (36.8%)
B-6	Number of drug-related incident events in hospital wards	Outcome	-	9 (11.8%)
C: Infection control				
C-1	Incidence of methicillin-resistant *Staphylococcus aureus* infection and carriage	Outcome	-	22 (28.9%)
C-2	Incidence of patients positive for *Clostridium difficile *toxin	Outcome	-	14 (18.4%)
C-3	Incidence of surgical site infection	Outcome	-	11 (14.5%)
C-4	Amount of rubbing hand alcohol used (wards)	Process	-	20 (26.3%)
C-5	Blood culture rates when broad-spectrum antimicrobials are used	Process	-	24 (31.6%)
D: Cancer care				
D-1	Five-year survival rate after cancer surgery	-	-	4 (5.3%)
D-2	Post-operative hospital stay for patients with gastrointestinal cancer	-	-	1 (1.3%)
D-3	Post-operative hospital stay for patients with lung cancer	-	-	2 (2.6%)
D-4	Number of postoperative hospital deaths	Outcome	-	2 (2.6%)
D-5	Percentage of emergency reoperation within 48 hours after surgery in hospitalized surgical patients	-	-	5 (6.6%)
E: Radiology/examination				
E-1	Percentage of radiologists completing CT and MRI reading reports by the next business day	Process	-	6 (7.9%)
E-2	Percentage of reading reports completed	Process	-	9(11.8%)
E-3	Number of histopathological diagnoses	Process	-	14 (18.7%)
F: Multidisciplinary team				
F-1	Number of patients with cancer instructed by doctors, nurses, etc.	Process	-	6 (7.9%)
F-2	Percentage of patients with diabetes and chronic kidney disease receiving nutritional management	Process	-	18 (23.7%)
F-3	Rate of care for patients at high risk of delirium	-	X	14 (18.4%)
G: Nursing				
G-1	Number (%) of bedsore high-risk patients cared	Process	-	12 (15.8%)
G-2	Pressure ulcer incidence rate	Outcome	X	61 (80.3%)
H: Community medical cooperation				
H-1	Number of cases of use of regional medical cooperation pass (five major cancers)	-	-	3 (3.9%)
H-2	Rate of hospitalization/discharge support	-	-	13 (17.3%)
I: Patient support (human rights protection)				
I-1	Rate of physical restraint of persons aged ≥20 years	Process	-	15 (19.7%)
I-2	Number of reports to and consultations with clinical ethics committees	Process	-	5 (6.6%)
I-3	Number of medical record disclosures (procedural disclosures)	Process	-	12 (15.8%)
I-4	Number of consultations with patients, families, etc.	Process	-	13 (17.1%)
I-5	SCC homepage: number of patient support/consultation visits	Process	-	1 (1.3%)
I-6	MSWs: rates of MSW intervention for patients transferred to other hospitals and institutions	-	-	4 (5.3%)
J: Patient convenience				
J-1	Percentage of respondents who would recommend the hospital to their friends in a patient survey	-	-	17 (22.4%)
J-2	Percentage of ambulant patients waiting for at least 1 hour before seeing a doctor	-	-	8 (10.5%)
J-3	Average outpatient waiting time	Process	-	14 (18.4%)
J-4	Waiting time for medicine	Process	-	0 (0.0%)
K: Preventive medicine				
K-1	Influenza vaccine immunization coverage among employees	Process	-	25 (32.9%)
K-2	Nonsmoking rate among employees	Process	-	4 (5.3%)
L: Human resource development				
L-1	Specialty/certified nurses per 100 nurses and those who have completed specific training	Structure	-	10 (13.2%)
L-2	Specialty/certified pharmacists per 100 pharmacists	Structure	-	6 (7.9%)
L-3	Specialty/certified medical advisors per 100 physicians	Structure	-	6 (7.9%)
L-4	Number of supervisors per resident doctor	Structure	-	6 (7.9%)
